# Prototype-Based Classifiers and Vector Quantization on a Quantum Computer—Implementing Integer Arithmetic Oracles for Nearest Prototype Search

**DOI:** 10.3390/e28020229

**Published:** 2026-02-16

**Authors:** Alexander Engelsberger, Magdalena Pšeničkova, Thomas Villmann

**Affiliations:** 1 Saxon Institute for Computational Intelligence and Machine Learning, University of Applied Sciences Mittweida, 09648 Mittweida, Germany; eardev@proton.me (A.E.);; 2 Bernoulli Institute for Mathematics, Computer Science and Artificial Intelligence, University of Groningen, 9712 CP Groningen, The Netherlands; 3 Faculty of Mathematics and Computer Science, Technical University Bergakademie Freiberg, 09599 Freiberg, Germany

**Keywords:** vector quantization, quantum machine learning, prototype-based learning

## Abstract

The superposition principle in quantum mechanics enables the encoding of an entire solution space within a single quantum state. By employing quantum routines such as amplitude amplification or the Quantum Approximate Optimization Algorithm (QAOA), this solution space can be explored in a computationally efficient manner to identify optimal or near-optimal solutions. In this article, we propose quantum circuits that operate on binary data representations to address a central task in prototype-based classification and representation learning, namely the so-called winner determination, which realizes the nearest prototype principle. We investigate quantum search algorithms to identify the closest prototype during prediction, as well as quantum optimization schemes for prototype selection in the training phase. For these algorithms, we design oracles based on arithmetic circuits that leverage quantum parallelism to apply mathematical operations simultaneously to multiple inputs. Furthermore, we introduce an oracle for prototype selection, integrated into a learning routine, which obviates the need for formulating the task as a binary optimization problem and thereby reduces the number of required auxiliary variables. All proposed oracles are implemented using the Python 3-based quantum machine learning framework PennyLane and empirically validated on synthetic benchmark datasets.

## 1. Introduction

As quantum computing nears commercialization, various research domains are emerging that complement hardware advancements with theoretical foundations, demonstrating the potential of quantum mechanics for computational tasks. One such field is quantum machine learning (QML) [1], where researchers seek quantum-enhanced approaches for processing large-scale datasets. Many current QML approaches utilize parameterized quantum circuits optimized via classical optimization schemes [2,3,4]. While these approaches effectively translate neural network architectures into the quantum realm, they face significant hurdles. Variational circuits often suffer from optimization landscapes that are flat, known as “barren plateaus” [5], and they cannot natively benefit from classical backpropagation [6]. Furthermore, like their classical counterparts, they often function as black boxes, lacking inherent interpretability [7].

Given the resource constraints of the current Noisy Intermediate-Scale Quantum (NISQ) era, algorithms with low computational complexity offer a distinct advantage. Prototype learning remains a compelling candidate due to its small computational footprint. Derived from *k*-Nearest Neighbors (*k*-NN), it relies on fundamental distance calculations. Unsupervised variants, such as the nearest centroid scheme (k-means clustering), have already been explored in the quantum context [8,9]. In the supervised domain, Learning Vector Quantization (LVQ) implements a classification scheme based on the nearest prototype principle [10].

In this work, we examine the synergy between quantum computing and prototype-based learning, such as LVQ. Quantum computing algorithms are largely defined by their data encoding strategy, which maps classical inputs to qubit states. Vector quantization-based quantum algorithms usually use an encoding known as amplitude encoding, where data is stored within the continuous amplitudes of a quantum state. This allows for high precision using a small number of computational units and an exponentially growing number of amplitudes based on the number of qubits. This strategy has been extensively theorized, particularly regarding kernel learning and Support Vector Machines (SVM) [11], as well as our own previous explorations of quantum-ready vector quantization [12,13].

However, while amplitude encoding is storage-efficient, performing non-linear distance calculations and comparisons between amplitudes is computationally challenging and often requires post-selection or expensive transformations between representations [14]. To address this, we propose utilizing basis encoding to leverage explicit quantum arithmetic for exact distance calculations. By employing basis encoding, we focus on leveraging quantum search routines, both unsorted and combinatorial, for prototype learning. We introduce quantum circuits that function as oracles within quantum search routines, which include explicit distance calculations. These oracles are optimized using variational methods, specifically the Quantum Approximate Optimization Algorithm (QAOA). To avoid overhead from the transformation of the problem statement into Hamiltonians, which introduce slack variables, we use an extension called program-based QAOA [15]. Additionally, we explore the use of Grover’s algorithm based on the same distance-calculating oracles.

We demonstrate both arithmetic-based approaches and confirm their validity through simulations. This paper is organized as follows: First, we introduce the theoretical foundations of vector quantization, quantum search, and quantum arithmetic. Next, we construct oracle circuits for specific prototype learning subtasks. Finally, we present proof-of-concept simulation results. Our goal is to provide a concrete implementation of these theoretical concepts and bridge the gap between quantum computing and prototype learning left by previous amplitude-encoding-centric research.

## 2. Vector Quantization

Learning Vector Quantization (LVQ) is a supervised machine learning algorithm [10]. Given a set of training samples $D={\left\{\left(\xi_{\mu },y_{\mu }\right)\right\}}_{\mu =1}^{N}$ with positions $\xi_{\mu }\in R^{n},\forall \mu$ and target labels $y_{\mu }\in C=\left\{1,\ldots ,N_{C}\right\},\forall \mu$. LVQ adjusts a set of labeled prototypes $W={\left\{\left(w_{\mu },c_{\mu }\right)\right\}}_{\mu =1}^{M}$ with positions $w_{\mu }\in R^{n},\forall \mu$ and target labels $c_{\mu }\in C,\forall \mu$. After training, a Nearest Prototype Classifier (NPC) $C_{\mathrm{NPC}}=\left(W,d\right)$ is obtained, where the prediction ${\hat{y}}_{j}=c_{\omega_{j}}$ is determined by the nearest prototype rule:

$$\omega_{j}={\mathrm{argmin}}_{i=1}^{M}d\left(\xi_{j},w_{i}\right),$$

which depends on the dissimilarity *d*. A common training scheme selects the prototypes such that the overall classification error

$$\sum_{j=1}^{N}\left[y_{j}\neq {\hat{y}}_{j}\right]$$

is minimized [10]. However, there are multiple training schemes that result in a nearest prototype classifier model [10,16,17].

Unsupervised vector quantization for representation learning and clustering is dominated by prototype-based (fuzzy) c-means variants and respective neural network vector quantizers, such as self-organizing maps and neural gas [10,18,19]. They all represent data by the most similar prototype according to the nearest prototype rule.

In general, vector quantization algorithms can be categorized with respect to the search space for the prototype positions. If prototypes are selected from the training sample itself and therefore $\forall i:(w_{i},c_{i})\in D$, it is categorized as a prototype selection scheme or a median variant [20,21,22]. When prototypes are generated without restrictions within the usually continuous data space of the training samples, e.g., $w_{i}\in R^{n}$, this method is called a prototype replacement scheme [23].

In the context of classical prototype-based classification learning, the replacement strategy, such as in LVQ, is often the preferred approach because selection strategies lead to the formation of combinatorial hard optimization problems, as demonstrated in [24]. Otherwise, prototype replacement strategies often follow minimization schemes of continuous cost functions, such as in generalized LVQ (GLVQ) [16] for classification or in unsupervised neural gas [19]. These functions are then optimized using gradient descent or similar strategies, allowing us to find approximations of the optimum.

According to these simple but intuitive learning and decision rules, unsupervised vector quantization schemes, as well as nearest prototype classifiers, belong to the family of interpretable machine learning models. A model becomes interpretable if its decision process can be understood by experts based on the model design given prior to the training itself [7].

In the context of quantum computing, this paper emphasizes the two essential ingredients of nearest prototype classifiers, which are both elementary for model decisions and their respective interpretations: the use of prototype positions as references and the choice of the dissimilarity measure. However, GLVQ variants use a parameterized dissimilarity $d_{\theta }$ with a vector of parameters θ for improved classification performance, which replaces the standard static dissimilarity *d* [17]. These dissimilarity parameters can be optimized during training, alongside the prototype positions, increasing the capabilities and interpretability of the vector quantization approach [25].

Being limited to replacement strategies can present significant limitations. The requirement of continuity of the cost function imposes constraints on the dissimilarity *d* and therefore the dataset; for instance, when the samples $\xi_{i}$ are not confined to a space such as $R^{n}$. In these cases, the samples might be any abstract object, such as molecules represented by graphs, necessitating embedding them into a continuous space. Such an embedding is crucial to facilitate the implementation of a replacement scheme but usually introduces distortions into the data, which affect the training and prediction quality. In the graph example, a possible embedding can be reached by mapping the dissimilarities to *n* specific examples called sensors into a response space $R^{n}$ [26]. A selection approach would be the natural choice in such a case, as it can use any dissimilarity defined on the abstract object set. Moreover, it does not result in prototypes that have no representation in the original data space, which would harm the interpretability of the trained model weights. A potential candidate for quantum advantages in prototype-based machine learning could be the efficient application of selection schemes for non-vectorial data.

In general, selection schemes lead to NP-hard optimization problems that are also valid for prototype-based classification learning. This has been proven by rewriting the prototype selection scheme as a binary optimization problem and showing that it is equivalent to the dominating set problem [24]. The authors call their model optimal p-prototypes nearest-neighbor (p-PNN) and formulate the “perfect classification problem” as finding the minimum of

$$\begin{array}{ccc} \mathrm{maximize}\; & \sum_{i\in I}z_{i}\\ \mathrm{subject}\;\mathrm{to}\; & \sum_{s\in w_{c}}x_{s}\geq 1 & \forall c\in C\\ \; & \sum_{s}x_{s}=p\\ \; & z_{i}\leq \left(1-x_{t}\right)+\sum_{s\in w_{c(i)}\cap w_{it}}x_{s} & \;\;\;\;\forall i\in I,t\notin w_{c(i)}\\ \; & x_{s}\in \left\{0,1\right\}\\ \; & z_{i}\in \left\{0,1\right\}\; \end{array}$$

being zero, which is identical to identifying a set of *p* prototypes in our search space that classify all training data points correctly using the nearest prototype classification rule. This quadratic optimization problem can be transformed into a Quadratic Unconstrained Binary Optimization (QUBO) of the form

$$y=\sum_{i,j<i}A_{ij}x_{i}x_{j}+\sum_{i}b_{i}x_{i}$$

for binary variables $x_{i}$ and weights $A_{ij},b_{i}$, standard strategies of quadratic programming are used.

## 3. Quantum Computing

Quantum computing is the utilization of quantum properties, primarily superposition, entanglement, and interference, to process data. The aim is to design algorithms that are likely intractable on classical computing machines. Research typically distinguishes between gate-based computing, which employs discretized quantum circuits analogous to classical logic, and adiabatic computing, which operates in an analog fashion by encoding problems into the ground state of a specific Hamiltonian. A short introduction to the mathematics of quantum computing is presented in Appendix A. Both paradigms are capable of addressing binary optimization tasks; for instance, the Quantum Approximate Optimization Algorithm (QAOA) serves as a gate-based discretization of the adiabatic approach. This work proposes shifting the classical transformation of optimization problems from a pre-processing step to arithmetic routines explicitly evaluated within the gate-based formalism.

A quantum computer is rarely envisioned as a standalone device. Instead, the proposed computer architectures use the quantum computer as a specialized subdevice of a classical computer, a quantum processing unit (QPU). The classical part of the computer loads the data from a classical source, builds a quantum circuit for gate-based devices or a schedule for an adiabatic device, and sends them to the QPU. After processing, the QPU returns the measurement results that were requested. A classical post-processing step calculates the final results or repeats the process as part of an optimization loop. One common post-processing technique is the approximation of a probability by counting multiple executions (shots) of the same circuit and calculating the relative frequency of each outcome.

## 4. Binary Data Representations

Here, we look at basis encoding, which is a way to load the superposition of binary strings into a quantum computer. Binary strings can be used in multiple ways to represent numerical data. We will use integer numbers later, as they require much simpler arithmetic operations than floating-point numbers, which are a representation of real numbers. However, in general, a sufficiently large quantum computer would be capable of processing any binary data, as it is a universal computer. The subsequently introduced algorithm requires two kinds of data representations to represent negative integer numbers: sign-magnitude and two’s complement. The commonly used two’s complement notation is a smart way to store signed integer numbers, as it allows subtraction by using the same addition logic designed for unsigned integers. This is achieved by adding another bit that indicates the sign of the number. However, unlike the sign magnitude notation, where the original bits in the string $i_{N-1}i_{N-2}\ldots i_{0}$ represent the magnitude as an unsigned integer

$$i=-1^{i_{N-1}}\sum_{k=0}^{N-2}i_{k}2^{k},$$

the sign bit in two’s complement is applied as follows:

$$i=-i_{N-1}2^{N-1}+\sum_{k=0}^{N-2}i_{k}2^{k}.$$

Note that we need to add one additional qubit in sign-magnitude notation to store all values of a two’s complement number because the sign-magnitude representation has two zeros and cannot represent the smallest value of the two’s complement representation with the same number of bits.

## 5. Quantum Search and Optimizers

Two classes of quantum algorithms will be useful in prototype learning: search algorithms and optimization algorithms. Both algorithms look for elements x∈X of a set called the search space. Search algorithms require an indicator function f(x)∈{0,1} that marks positive elements and searches for elements that fulfill f(x)=1. An optimization algorithm takes a cost function g(x)∈R and is designed to find ${\mathrm{argmin}}_{x\in X}g\left(x\right)$. We will look at these different types of algorithms: search with amplitude amplification, optimization with search algorithms, and optimization with variational circuits.

### 5.1. Search with Amplitude Amplification

There are known advantages in search tasks that a quantum search algorithm can provide for the computational problem of unordered search. They require a set of input values $X=(x_{1},\ldots ,x_{n})$ and an indicator function

$$f_{A}\left(x\right)=\left\{\begin{array}{cc} 1, & \mathrm{if}\;x\;\mathrm{has}\;\mathrm{property}\;A\\ 0, & \mathrm{if}\;x\;\mathrm{has}\;\mathrm{not}\;\mathrm{property}\;A \end{array}\right.$$

for an arbitrary property called *A*. Without loss of generality, we assume that $\left|X\right|=n=2^{m}$ for some m∈N, which simplifies the subroutines that initialize the quantum states. The unordered search is the task of finding one or all $x^{*}\in X$ such that $f(x^{*})=1$, without any additional information or structure on the search space X. Finding an element in the unordered case requires visiting all elements and checking for property *A* until a target element is detected. Although this is still valid for a quantum computer in some sense, there are strategies that could be seen as checking property *A* on all elements in a single step. However, they require some additional logic to read the solution back from the quantum computer to overcome the problem of the wave function collapsing during measurement.

Finding elements in an unordered set can be performed more efficiently than the best-known classical search algorithms using Grover’s search [27]. A quantum register with *m* qubits is initialized in an equal superposition of the indices of X

$$\frac{1}{\sqrt{2^{m}}}\sum_{i}|i\rangle .$$

Given this superposition of the indices, the Grover search algorithm increases the probability of measurement for the indices *i*, where the corresponding $x_{i}$ results in $f_{A}\left(x_{i}\right)=1$. This is done using an oracle $O_{A}$, a routine that flips the face of all *i*’s that are marked by $f_{A}$.

For now, we assume that a circuit $F_{A}$ is given that implements $F_{A}|x\rangle |0\rangle \to |x\rangle |f_{A}\left(x\right)\rangle$. From this circuit, a routine can be constructed that flips the face of all elements with $F_{A}\left(x_{i}\right)=1$

$$O_{A}|x\rangle \to {\left(-1\right)}^{f_{A}\left(x\right)}|x\rangle .$$

This is done by a phase kickback that interacts with another qubit after applying $F_{A}$.

$$|x\rangle |0\rangle |-\rangle \to^{F_{A}}|x\rangle |f_{A}\left(x\right)\rangle |-\rangle \to^{CNOT}|x\rangle ⊗-|f_{A}\left(x\right)\rangle |-\rangle \to^{F_{A}^{†}}-|x\rangle |0\rangle |-\rangle ,$$

where the CNOT gate flips the sign based on the function’s output.

Because a phase change does not affect any measurable property of the quantum system, this step alone is not useful. Invoking interference effects is necessary, using the Grover diffusion operator *D* to invert each amplitude centered around the mean. This diffusion acts as

$$D\sum_{j}\alpha_{j}|i\rangle \to \sum_{j}\left(2\mu -\alpha_{j}\right)|i\rangle ,$$

with $\mu =\sum_{j}\alpha_{j}$ as the mean amplitude.

These two steps of applying the oracle and then the diffusion must be repeated. The ideal number of repetitions is

$$\left\lfloor \frac{\pi }{4}\sqrt{\frac{2^{m}}{k}}\right\rfloor ,$$

with *k* being the number of elements $x_{i}$ with $f_{A}\left(x_{i}\right)=1$. If *k* is not known, strategies exist to determine it or to wrap the search in another loop, guessing *k* [28].

### 5.2. Optimization with Search Algorithms

In a nearest prototype classifier, an ideal indicator function marks the minimum element, and we could use Grover search inside the nearest prototype rule. However, such an oracle depends on all elements in the search space, and Grover’s search requires the oracle to be executable independently for each state. By searching for states below a shrinking threshold, we can work around this limitation. The basic idea is to start from a random point $x_{0}$ and calculate the value $y_{0}=d\left(x_{0}\right)$ for the target function *d* that we want to minimize. The oracle is then designed so that

$$f_{y_{0}}\left(x\right)=\left\{\begin{array}{cc} 1, & \mathrm{if}\;d\left(x\right)<y_{o}\\ 0, & \mathrm{else} \end{array}\right..$$

This oracle is used in a Grover search algorithm to increase the probability of finding an element below the threshold. After a random measurement of an $x_{1}$ where $d\left(x_{0}\right)>d\left(x_{1}\right)$, the oracle is updated with the new threshold $y_{1}=d\left(x_{1}\right)$. The result is a chain of elements with a shrinking value. This minimization approach and some theoretical analysis, such as the number of iterations, were published by Dürr and Høyer [29].

### 5.3. Optimization with Variational Circuits

A large field in quantum computing is variational quantum computing (VQC). The term variational circuit describes a parameterized function that takes a set of parameters and returns the instructions for a quantum circuit. A cost function based on measurements inside such a circuit is optimized by ’varying’ the parameters. This is usually done by using a classical optimization scheme in a hybrid computing environment. The most prominent example of VQC is a quantum neural network.

A variational algorithm for solving combinatorial problems is the Quantum Approximate Optimization Algorithm (QAOA) [30] and its variants. QAOA expects the optimization problem to be given as a Quadratic Unconstrained Binary Optimization Problem (QUBO). From this, a Hamiltonian is created that is used within a parameterized circuit for time evolution. The parameters are then optimized using an optimization scheme with the goal of increasing the probability of measuring closer to optimum solutions. The QAOA was originally designed to solve combinatorial problems like maximal cuts in graphs. It can be seen as a discretized variant of adiabatic optimization; hence, both share a common development, with concepts being transferred between them. For this work, two concepts are of greater interest: the optimization of a cost function and the use of adiabatic concepts, and hence QAOA for unstructured search. It can be shown that circuits derived from the original QAOA can optimize search problems with a similar complexity to the amplitude amplification method used in Grover’s algorithm [31].

The QAOA circuit is built from two alternating components: a cost Hamiltonian and a mixing Hamiltonian. The cost Hamiltonian is specific to an optimization task; it can be derived from a QUBO cost function by replacing binary variables $x_{i}$ with an *Z* operator acting on the qubit *i*. This requires the problem to be available as Hamiltonian or QUBO in the first place and is a limiting factor that we will weaken in a later step. The projective measurement of this operator returns the cost function value for a binary input encoded in the qubits. The mixing Hamiltonian, on the other hand, is usually chosen from a small set of known mixers. Common mixers are the sum of *X* operators on each qubit and a variant of the diffusion step of Grover’s search.

## 6. Prototype Selection with Ising Model

The selection of prototypes on a quantum computer can be done by transforming a binary problem statement into a Hamiltonian [12]. This transformation uses the so-called Ising Spin Model as an intermediate representation. A spin variable has two states, but instead of using binary values 0,1, it can have the states −1,+1. Quantum computers can exhibit the same behavior with values that are measured as −1 or +1. A given Ising model can be simply rewritten as a Hamiltonian, which, in turn, can be evaluated on a quantum device. There are multiple approaches to find the optimal or approximately optimal assignments of the spin variables.

Quantum annealing is a strategy for finding the minimum eigenvalue (ground state) of a problem Hamiltonian. This is done by transforming a quantum system initialized in the known ground state of a simpler Hamiltonian that acts on the system. By slowly transforming the Hamiltonian acting on the system from this initial one into the problem Hamiltonian, the system stays in the ground state of the driving operator. If this is done slowly enough, the system will be in the ground state of the problem Hamiltonian and hence optimize the problem task. The system can then be measured to obtain the solution.

In some state-of-the-art quantum algorithms, it is necessary to convert the initial problem statement into an Ising model to optimize it. There are many standard Ising formulations for common problems [32], but they introduce many slack variables. In numerous cases, it is more natural to express the target value as a circuit implementing a mathematical function and to use it within an optimization algorithm. In Section 7, strategies are provided that transform target functions into quantum circuits that can act as a subroutine within an optimization scheme. This is possible with the recent extension of QAOA to accept arbitrary cost circuits, program-based QAOA (Prog-QAOA) [15].

Prog-QAOA distills the fact that QAOA depends on the phase change that the cost Hamiltonian introduces into the system into an explicit phase gate. This phase gate is controlled by the output of the cost circuit, which is specific to the optimization problem. Hence, it can waive the requirement of encoding the problem as QUBO, and by doing so, it removes the drawbacks of this transformation. In particular, the encoding of constraints, which occurs in the creation of the QUBO formulation from an arbitrary integer programming statement, can introduce numerous additional variables and, therefore, increase the required number of qubits. In prog-QAOA, these constraints are also written as functions that are summed with the original cost using a sufficiently large weight, and then the total cost function is implemented as a quantum routine. The mixer Hamiltonian of the QAOA is not derived from the cost function but is chosen from a set of simple-to-implement Hamiltonians so that it matches the general structure of the cost function. Prog-QAOA can be based on the same mixer as the original QAOA and its extensions; therefore, the main task is to design the cost routines using quantum arithmetic.

## 7. Quantum Arithmetic Routines

Quantum arithmetic routines are circuits that calculate functions in a quantum register, whose state represents a number. A key mathematical function in prototype learning is the calculation of the distance between a query vector and each prototype. For this, we introduce a subroutine that can evaluate a specific distance function between two integer vectors. One variant returns the taxi-cab or Manhattan distance, while the other returns the squared Euclidean distance. These distances were selected not only because they have a simple arithmetic structure but also because the squared Euclidean distance is one of the most common distances in prototype learning.

We construct the two distance circuits from smaller arithmetic routines that are then combined. There is always the possibility of taking a known classical logic circuit that solves a task and converting it into a quantum circuit by transforming it into a reversible logic circuit and mapping it to a quantum circuit [33]. In this section, we will focus on quantum computer-specific implementations that can replace these synthesized circuits. These subroutines [34,35] are collected and implemented in the PennyLane Python framework [36] and are referenced in its documentation. Another collection of arithmetic routine implementations is Qualtran [37]. The reader is referred to the aforementioned references for details on the current state-of-the-art implementations. Given the dynamic nature of the field, these technical specifics are subject to ongoing refinement and are not addressed in further detail in this paper.

### 7.1. Single Register Arithmetic

For a single register, we first need the basic arithmetic operations of addition and subtraction of a constant value:

$${\mathrm{AddConst}}_{M}\left(k\right)\left[|m\rangle \right]=|m+k\;\mathrm{mod}\;M\rangle .$$

Because subtraction can be accomplished through addition using two’s complement notation, we only need to find a routine that can add a constant to a quantum register in basis encoding. The current state-of-the-art circuits for modular addition are either based on the Quantum Fourier Transformation for modular addition [35] or have been optimized to require fewer instances of a specific quantum gate called the T-gate [38]. These T-gates are challenging to implement physically; they also introduce a type of hardness that cannot be simulated efficiently on a classical computer.

The second group of operations is related to the handling of negative numbers. First, we need a way to determine the absolute value f(x)=|x| of a signed number *x*. By converting to the sign-magnitude notation, this operation becomes reversible:

$$\mathrm{Sign}\text{-}\mathrm{Magnitude}\left[{|x\rangle }_{N}{|0\rangle }_{1}\right]={||x|\rangle }_{N}{|\mathrm{sign}(x)\rangle }_{1}.$$

The approach to synthesize this conversion from a classical method requires gates that are considered expensive in the current generation of hardware; alternatives have been proposed [34]. A routine to convert the sign-magnitude into the two’s complement representation is implemented in Qualtran [39]. Its inverse could be used to find the absolute value.

The final routine we need for a single register is the sign inversion or negation using two’s complement:

$$\mathrm{Inv}\left[|x\rangle \right]=|-x\rangle .$$

Given the addition routine, this is achieved by flipping all bits and adding 1 using AddConst_M_(1).

### 7.2. Multiple Register Arithmetic

For multiple registers, we also require basic arithmetic operators, such as addition; however, instead of adding a constant value to a quantum value, the values of two quantum registers are combined:

$${\mathrm{Add}}_{M}\left[|m\rangle |n\rangle |0\rangle \right]=|m\rangle |n\rangle |m+n\;\mathrm{mod}\;M\rangle .$$

Because the output value is stored in a third register, this is called out-of-place addition. This can be done by a controlled variant of in-place addition [35]. The out-of-place polynomial operation [40] necessary for the squared Euclidean distance is likewise based on the Fourier transform.

## 8. Vector Quantization Using Quantum Arithmetic

For Grover’s search and prog-QAOA, we require an oracle circuit that marks inputs w if they fulfill $f_{A}\left(w\right)=1$. A special case of such an oracle occurs when Grover’s search is used to find a minimum, where an oracle is utilized that marks elements below a threshold, i.e., *A* is an expression of the form A=[g(w)≤θ], for an arbitrary function *g* and a threshold θ. We start with a simple oracle where g(x)=x and show how a threshold can be implemented using quantum arithmetic; then, we introduce an oracle where *g* is a distance function expressed by quantum gates. The third oracle introduced here uses calculated distances to mark prototype pairs that accurately classify a query vector.

### 8.1. Threshold Oracle

Before we use quantum arithmetic circuits to calculate distances, we first look at how we can use these distances to determine the winner. Having the ability to mark elements below a threshold is required to apply the Dürr-Høyer optimization scheme. For this, we construct an oracle that marks all elements of a list $\left[d_{1},\ldots ,d_{n}\right]$ that are below a threshold θ. For simplicity, we assume that all $d_{i}$ and θ are integers. As input, we take a quantum register that can encode the positions in the list 1,…,n; hence, the input to the circuit is

$$\sum_{i=1}^{n}w_{i}|i\rangle ,\;$$

with complex weights $w_{i}$ for each index. Using this input, the positions $d_{i}$ are loaded into a sufficiently large working register using a QROM routine. After this step, the indices are entangled with their corresponding values

$$\sum_{i=1}^{n}w_{i}|i\rangle |d_{i}\rangle .\;$$

The threshold is implemented by employing the in-place adder subroutine illustrated earlier on the data stored in the working register, which modifies the weight distribution to

$$\sum_{i}w_{i}|i\rangle |d_{i}-\theta \rangle .$$

The sign bit of the second register almost contains the output of the oracle; it is one when $\theta >d_{i}$ and zero otherwise. By a controlled NOT operation on a flag qubit prepared as |1〉, we obtain the desired output

$$\sum_{i}w_{i}|i\rangle |d_{i}-\theta \rangle |[d_{i}\leq \theta ]\rangle .\;$$

**Figure 1:**
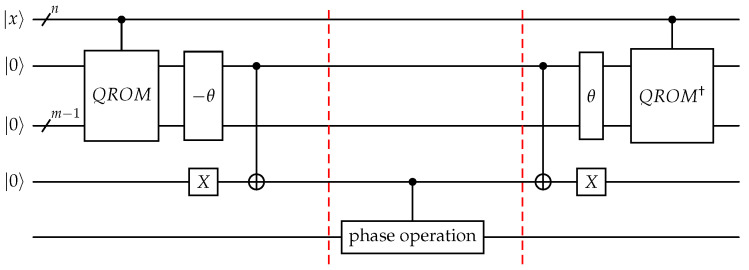
Circuit of the threshold oracle. The red dottet lines seperate forward pass and uncomputation from the phase shift.

This value can then be used for a phase kickback or phase separation, after which the flag and working registers can be freed using uncomputation. The whole circuit is shown in Figure 1.

### 8.2. Close-Range Prototype Oracle

The previous oracle assumed a list of distances as input, marking the elements below a threshold. With the presented arithmetic subroutines, we can extend this oracle so that it uses quantum parallelism to calculate distances in a Single Instruction Multiple Data (SIMD) manner. We first introduce a subroutine for calculating two types of distances and later combine them with the simple threshold oracle into the close-range prototype oracle.

The constructed subroutine will calculate the distance between a data vector $x={\left[x_{1},\ldots ,x_{n}\right]}^{T}$ and a prototype $w={\left[w_{1},\ldots ,w_{n}\right]}^{T}$. The first example is the squared Euclidean distance

$$d_{E}^{2}\left(w,x\right)=\sum_{i}{\left|w_{i}-x_{i}\right|}^{2},$$

and the second is the Manhattan (taxicab) distance

$$d_{M}\left(w,x\right)=\sum_{i}\left|w_{i}-x_{i}\right|.\;$$

Both require only the aforementioned arithmetic operations.

The system starts in the following state:

$$|w\rangle =|w_{1}\rangle |w_{2}\rangle \ldots |w_{n}\rangle |0\rangle =\underset{i}{⨂}|w_{i}\rangle ⊗|0\rangle$$

for an integer vector w with *n* dimensions. The register has a width of nl+1, where l is the word length chosen to be large enough to store the data points and all possible results. The vector register is divided into *n* feature registers of width *l* and one output qubit. Additional helper registers are required that are used during calculations to store intermediate results or information needed for reversible computing. It is important to keep them in mind, as they increase the required number of qubits on the quantum device.

**Figure 2:**
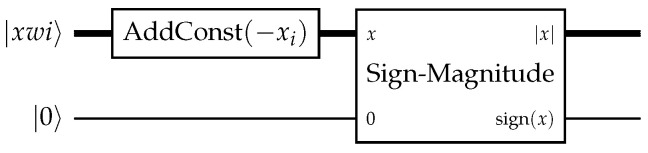
Absolute Difference Operator.

Having the position of the point w loaded, the distance to x can be calculated with $x_{i}$ embedded in the circuit as constants. For each feature register, we calculate $|w_{i}-x_{i}|$ in parallel using the absolute difference operator in Figure 2. This is done by first applying an in-place addition routine with the parameter $-x_{i}$ to the register. Then, one of the possible circuits to calculate the sign and magnitude representation of an integer from the 2’s complement is applied. Leaving the register in the state

$$|\psi \rangle =\underset{i}{⨂}[||w_{i}-x_{i}|\rangle ⊗|\mathrm{sign}(w_{i}-x_{i})\rangle ]⊗|0\rangle .$$

Storing the sign is necessary to reverse this operation in the following uncomputation. However, since we are not interested in it, we will skip it in the following steps to make the expressions shorter.

If we want to calculate the taxicab distance, we use a combination of out-of-place addition routines to sum up all values from the previous step:

$$|\psi \rangle =\underset{i}{⨂}||w_{i}-x_{i}|\rangle ⊗|\sum_{i}\left|w_{i}-x_{i}\right|\rangle =\underset{i}{⨂}||w_{i}-x_{i}|\rangle ⊗|d_{0}\left(w,x\right)\rangle .$$

For the squared Euclidean distance, we can use the out-of-place polynomial operation [40]:

$$|\psi \rangle =\underset{i}{⨂}||w_{i}-x_{i}|\rangle ⊗|\sum_{i}{\left|w_{i}-x_{i}\right|}^{2}\rangle =\underset{i}{⨂}||w_{i}-x_{i}|\rangle ⊗|d_{2}^{2}\left(w,x\right)\rangle .$$

Despite the existence of square root approximations in quantum devices [41], their utilization is omitted due to their substantial computational demand and the fact that only the order of different distances is required in the nearest prototype decision. The square root function is order-preserving for positive integers and hence does not change the results for prototype competition.

The nearest prototype oracle takes a prototype position and a query position as input, as well as a threshold. Then it returns a value that indicates whether the prototype is closer to the query position than the threshold. Marking a value *d* as below a threshold θ requires an in-place addition and the use of two’s complements. When the value of θ is subtracted from *x*, the sign bit of the result is already the required output.

For the oracle, we start from an index value |i〉 and load the prototype position using a subroutine such as QROM into an empty register, leading to the state $|i\rangle |w_{i}\rangle$. Then we apply the distance subroutine for a data point x, creating the state:

$$|i\rangle |w_{i}\rangle |d(w_{i},x)\rangle .$$

Write $|i\rangle |d_{i}\rangle$ to keep things short.

Using another in-place addition routine to subtract the threshold θ, we get $|i\rangle |d_{i}-\theta \rangle$, where the sign bit of the data register $|d_{i}-\theta \rangle$ marks prototypes that are closer to x than the threshold.

### 8.3. Best Selection Oracle

**Figure 3:**
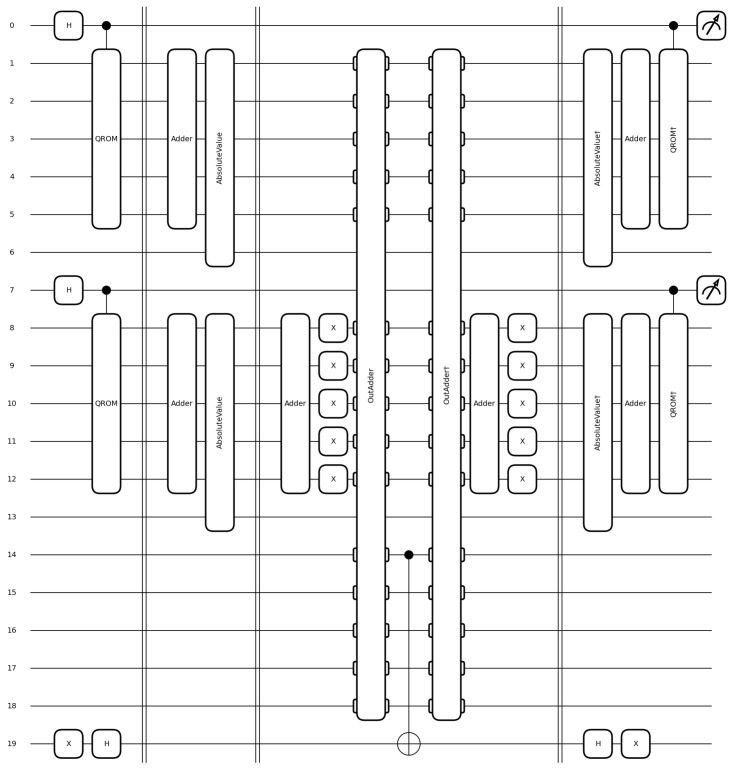
Circuit that implements the Best Selection Oracle for two prototypes per class and a data width of four. The vertical lines seperate the steps: data loading, distance calculation, distance comparison and uncomputation.

While the close-range prototype oracle is used in the prediction phase of prototype learning, the following oracle can be used to train a nearest prototype classifier. The best selection oracle shown in Figure 3 checks whether a given prototype selection will correctly classify a sample. Let $I_{0}$ and $I_{1}$ be the index sets of all candidate prototypes that have classes 0 and 1, respectively. The positions of the candidates are given as $x_{i},\forall i\in I_{0}\cap I_{1}$. Additionally, we can have $c_{0}$ and $c_{1}$ as the number of prototypes requested for the two classes. For simplicity, we set $c_{0}=c_{1}=1$ to keep the circuits in the introduction thinner. In this case, we expect the prototype to mark prototype pairs that correctly classify a sample ξ=(s,t),t∈{0,1}, using the nearest prototype rule and a distance *d*. The oracle is responsible for identifying all combinations of indices $\left(i_{0},i_{1}\right)\in I_{0}\times I_{1}$ that accurately classify the sample:

$$O_{\xi }\left(i_{0},i_{1}\right)=\left\{\begin{array}{cc} 1 & \mathrm{if}\;t=0\wedge \left[d\left(x_{i_{0}},s\right)<d\left(x_{i_{1}},s\right)\right]\\ 1 & \mathrm{if}\;t=1\wedge \left[d\left(x_{i_{0}},s\right)>d\left(x_{i_{1}},s\right)\right]\\ 0 & \mathrm{else}. \end{array}\right.$$

To implement the oracle as a circuit, we use the same subroutines presented in the close-range prototype oracle. For each class, the prototype positions are loaded based on the superposition in its index register. Then, the distance subroutine is used to calculate the distance of each prototype to the sample in parallel.

The superposition of all distances between the sample and the prototypes, grouped by class label, is then negated for the prototypes that have non-matching classes. After adding the values using an out-of-place adder [35] to a third register, the sign bit of the third register is the desired output.

## 9. Simulations

To demonstrate the practical feasibility and performance of the newly proposed methods, we now turn to a series of numerical simulations. All simulations have been developed, implemented, and executed using the PennyLane Python framework [36], which provides tools tailored for quantum and hybrid quantum-classical computations.

### 9.1. Oracles in Grover Search

We start with a numerical experiment that implements the proposed oracles within the Grover search framework and evaluates their effectiveness in solving prototype learning tasks. In these simulations, we consider a finite input set of four elements, exactly one of which is designated as the target. For this specific configuration, the target element can be located with unit success probability by performing a single iteration of the standard Grover amplitude amplification procedure; that is, in the absence of noise, the algorithm deterministically outputs the correct solution in this setting.

#### 9.1.1. Simulation 1

The first simulation evaluates, for each of the four specified prototypes, whether their distance to a given sample falls below a predefined threshold. The close-range prototype oracle (see Section 8.2) can be used within quantum minimization to find the prototype closest to a sample. We take $W=\left[-3,-1,4,6\right]\subset R^{1}$ as prototypes, ξ=2 as a sample, and a threshold θ=3 as our example. For the width of the arithmetic register, we took 4 bits, allowing it to represent numbers between −8 and 7; the index takes 2 bits to represent the four prototypes. Taking another qubit used in a sign-magnitude conversion and the qubit necessary for phase kickback, we find a total of 8 qubits required simultaneously.

#### 9.1.2. Simulation 2

The second simulation setting shows that Grover’s search and the best selection oracle (see Section 8.3) can be used to find a pair of prototypes that correctly classify a data point. The prototype candidates and the data point are selected from $R^{1}$, and the distance used is the absolute difference between the values, which corresponds to the Manhattan distance and the Euclidean distance in one dimension. Since there are 4 prototype candidates, two from each class, we have four possible pairs, where both classes are represented once. The positions are chosen in such a way that only one solution classifies the data point correctly, thereby obtaining the optimal Grover setting. We use the values from the previous simulation for the prototypes and the sample; the prototype −3 and −1 belong to the first class, 4 and 6 belong to the second, and the sample belongs to the first.

### 9.2. The Grover Search for Both Simulation Settings

Generally, Grover search acts on a register of unsigned integers that represent the indices of the elements in the prototype set. The search starts with an equal superposition within this register. Whenever there is an *n* such that there are $2^{n}$ inputs, preparing this superposition is simply done by the parallel application of *n* Hadamard gates. Otherwise, there are strategies using the quantum Fourier transformation to create an equal superposition of the relevant elements. The first step in both of the above simulations is the preparation of an equal superposition in the index register, and we prepare a phase kickback bit in the state |−〉.

In addition, in both simulations, the prototype positions are loaded by the QROM subroutine into a working register. For the threshold oracle, this is done on a single index working register pair; for the best selection oracle, this is done on two separate pairs, one for each class. As both simulations use the absolute difference as a distance, the routine to calculate this distance from the given sample is applied to the working registers that hold the prototype positions. After this step, the values of the index registers are entangled with the distance to the sample of the respective prototype.

Both oracles generate their results by computing differences, effectively performing a comparison between two values. The threshold oracle compares the value stored in the work register with a specified threshold using a subroutine that applies this threshold value. In the best selection oracle, a comparison between two work registers is performed by inverting the value of one register and then using an out-of-place adder to obtain the difference between the two registers. Both oracles encode their output in the sign bit of the difference.

As we have prepared a phase-kickback qubit in the |−〉 state, it can be used to turn the output qubit of the oracle into a phase flip of the target states. This is done by applying a controlled NOT gate between the output of the oracle and the phase-kickback qubit, which leads to the aforementioned phase kickback. After that, we proceed with the uncomputation of the oracle to clean up the working register, leaving us with only the phase flip. After applying the Grover mixer or diffusion on the index register, the iteration step is completed. By the design of the simulation, the state can be measured after this single iteration. Thus, in both cases, the simulation returns a 100% chance of measuring the desired solution.

## 10. Conclusions

In this study, we extended the theoretical framework of quantum prototype learning by incorporating strategies that rely on basis encoding. By moving away from the amplitude encoding conventionally used in Quantum Machine Learning (QML), we broadened the possibilities for realizing intrinsically interpretable machine learning models on quantum hardware. Basis encoding supports parallel processing of data stored in superposition, offering more control over distance computations than previous techniques based on state-overlap circuits. This design choice allows the use of arbitrary distance metrics and removes the need to approximate output values through repeated measurements.

Our investigation focused on concrete realizations of how quantum search and optimization algorithms can resolve core tasks within prototype-based classification. Specifically, we demonstrated that classification learning tasks can be mapped to quantum circuits that parallelize both distance computation for winner determination and the identification of prototype sets that accurately classify training vectors. We introduced two specialized oracles to achieve this: the Close-Range Oracle, which identifies prototypes within a defined threshold to enable Grover-based minimum search with its characteristic quadratic speedup, and the Best Selection Oracle, which identifies prototype combinations satisfying the nearest prototype rule. By utilizing the latter within the recently proposed program-based QAOA framework, we successfully circumvented the computationally expensive and qubit-intensive transformation of constraints into problem Hamiltonians.

In our simulations, we demonstrated that the constructed oracles behave as intended on minimal test instances. We successfully replicated the expected results with an implementation based on arithmetic operators provided by the PennyLane framework, thereby confirming the validity of arithmetic oracles within the frameworks of QAOA and Grover’s search. Future research should address more demanding real-world applications, which will likely necessitate more advanced implementation strategies and may also involve additional numerical (quantum) optimization procedures. Another open direction is to rigorously quantify the algorithmic complexity and compare the present results with those from our earlier work and classical approaches in general.

## Data Availability

All used datasets for the simulations are described inside the text.

## Appendix A. A Brief Introduction to Quantum Computing

The atomic unit of quantum computing, and hence the counterpart of the classical bit, is a qubit [42]. Its state is a linear combination

$$|\psi \rangle =a|0\rangle +b|1\rangle \;\;\mathrm{with}\;\;a,b\in {C:\;|a|}^{2}+{\left|b\right|}^{2}=1$$

of the two basis states,

$$|0\rangle =\left(\begin{array}{c} 1\\ 0 \end{array}\right),\;|1\rangle =\left(\begin{array}{c} 0\\ 1 \end{array}\right).$$

To store binary data on a device, we use *N* qubits that are combined into a quantum register:

$$\psi =\sum_{i=0}^{2^{N}-1}\psi_{i}|i\rangle =:|\psi \rangle \;\;\mathrm{with}\;\;\sum_{i=0}^{2^{N}-1}{\left|\psi_{i}\right|}^{2}=1.\;$$

Here we write

$$|i\rangle :=|b{\left(i\right)}_{0},\ldots ,b{\left(i\right)}_{n}\rangle :=|b{\left(i\right)}_{0}\rangle ⊗\cdots ⊗|b{\left(i\right)}_{N-1}\rangle$$

for a binary representation $b:i\mapsto {\left[0,1\right]}^{N}$.

The state of a quantum register can be considered a weighted distribution across all bit strings of length *N*, with the weight $\psi_{i}$ for the binary string b(i). This phenomenon of storing multiple states at once is commonly referred to as superposition. Of course, these binary strings can be interpreted differently, e.g., as integers in their binary notation or as floating-point numbers. The catch is that this superposition does not allow all states to be read from a quantum register. When measured, one of the states will randomly be chosen with probability $|\psi_{i}{|}^{2}$, and the system collapses into the state |i〉, losing all information about the weights before the measurement.

The change in the state of a quantum register is denoted as time evolution. Its behavior is described by the time-dependent Schrödinger Equation

$$i\hbar \frac{d}{dt}|\Psi (t)\rangle =\hat{H}|\Psi (t)\rangle ,$$

where $\hat{H}$ is a Hamiltonian operator. If the operator is constant over time, the time evolution becomes

|Ψ(t)〉=U(t)|Ψ(0)〉,

with $U\left(t\right)=e^{-i\hat{H}t/\hbar }$ being a unitary operator acting on the state.

An interesting trick with time evolution allows the use of the adiabatic theorem for a time-dependent Hamiltonian $\hat{H}\left(t\right)$ to solve computational problems [43]. We assume that $\hat{H}\left(t\right)$ can be decomposed into two time-independent Hamiltonians ${\hat{H}}_{A},{\hat{H}}_{B}$ as

$$\hat{H}\left(t\right)=\frac{T-t}{T}{\hat{H}}_{A}+\frac{t}{T}{\hat{H}}_{B}$$

with time 0≤t≤T, where *T* is a sufficiently large parameter based on the computational problem. Then, starting from |Ψ(0)〉 as the eigenvector of ${\hat{H}}_{A}$ corresponding to the lowest eigenvalue, the result |Ψ(T)〉 will be the eigenvector of ${\hat{H}}_{B}$ corresponding to the lowest eigenvalue. This can be used in optimization tasks, as we show later.

A more common form of using time evolution is the manipulation of quantum states using unitary operators that represent logic operations, called gates. As a simple example, we look at the PauliX operator that is given by the matrix

$$\sigma_{x}=\left(\begin{array}{cc} 0 & 1\\ 1 & 0 \end{array}\right).$$

When acting on |0〉, we obtain the following result

$$\sigma_{x}|0\rangle =\left(\begin{array}{cc} 0 & 1\\ 1 & 0 \end{array}\right)\left(\begin{array}{c} 1\\ 0 \end{array}\right)=\left(\begin{array}{c} 0\\ 1 \end{array}\right)=|1\rangle$$

and similar $\sigma_{x}|1\rangle =|0\rangle$. Hence, the Pauli X operator acts as a logical NOT gate on a single qubit. Note that in a superposition, it exchanges the weights between the outcomes, acting as a NOT gate on both states at the same time. Other gates can act on multiple qubits at once. A series of gates is called a quantum circuit, and a part of a circuit that applies a function is called a (sub)routine.

As mentioned above, when measured, a superposition collapses randomly depending on its weights. From a probability point of view, we recognize that the weights $-1\psi_{i}$ and $\psi_{i}$ are identical because the probability $|\psi_{i}|$ is not affected by the phase. This phase, which can be any normalized complex number, while not measurable, can affect the outcome of quantum gates through interference.

Quantum read-only memory (QROM) is a form of state preparation based on an address or index register. It can be used to load states based on a predefined set of states by an address register:

$$\mathrm{QROM}|i\rangle |0\rangle =|i\rangle |x_{i}\rangle .$$

The most basic variant of a QROM is a chain of so-called Select operators

$$\mathrm{Select}|i\rangle |\psi \rangle =|i\rangle U_{i}|\psi \rangle ,$$

where $U_{i}|0\rangle =|x_{i}\rangle$ is a combination of Pauli-X gates. Other approaches implemented in the PennyLane framework trade reduced circuit length for higher circuit width. We utilize both representations in the circuits shown in this work. Indices marking elements in an input set are stored in quantum registers that contain a weighted distribution between the values $i\in \{0,\ldots ,2^{N}-1\}$ given in unsigned integer notation. The number *i* is stored as the tensor product of the binary qubit states $|i\rangle =|i_{N-1}\ldots i_{0}\rangle =|i_{N-1}\rangle \ldots |i_{0}\rangle$ where $i=\sum_{k=0}^{N-1}i_{k}2^{k}$ and $i_{k}\in \left\{0,1\right\}$. To process data, we use data registers that store integer values $x\in \{-2^{N-1},\ldots ,0,\ldots ,2^{N-1}-1\}$ using 2’s complement notation.
